# A Comprehensive Review of Screening Methods for Ovarian Masses: Towards Earlier Detection

**DOI:** 10.7759/cureus.48534

**Published:** 2023-11-08

**Authors:** Shreya A Sahu, Deepti Shrivastava

**Affiliations:** 1 Obstetrics and Gynaecology, Jawaharlal Nehru Medical College, Datta Meghe Institute of Higher Education and Research, Wardha, IND

**Keywords:** emerging technologies, biomarkers, high-risk populations, screening methods, early detection, ovarian masses

## Abstract

Ovarian masses, ranging from benign cysts to malignant tumors, present complex diagnostic challenges in women's healthcare. Early detection of ovarian masses is paramount for improving patient outcomes, as delayed diagnoses often lead to advanced-stage disease with limited treatment options. This comprehensive review explores screening methods' current state, limitations, and emerging technologies to facilitate earlier detection. The limitations of existing screening methods, such as low sensitivity and specificity, underscore the need for improved early detection strategies. Imaging-based techniques, including transvaginal ultrasound, magnetic resonance imaging, and computed tomography, are vital in evaluating ovarian masses. However, the emergence of artificial intelligence (AI) and machine learning (ML) applications enhances the accuracy of image interpretation. Blood-based biomarkers, such as CA-125, have been the focus of research for ovarian mass detection. While CA-125 remains widely used, its limitations have prompted investigations into alternative serum biomarkers, including HE4 and miRNA, along with liquid biopsy and circulating tumor DNA. Ultrasound-based scoring systems, such as the risk of malignancy index (RMI), Ovarian-Adnexal Reporting and Data System (O-RADS), and guidelines from the International Ovarian Tumor Analysis (IOTA) group, provide structured approaches for classifying ovarian masses. These systems aid healthcare providers in clinical decision-making. Emerging technologies, such as liquid biopsy, AI, and proteomic/metabolomic approaches, offer promising avenues for enhancing early detection and risk assessment. Liquid biopsy provides noninvasive, real-time monitoring of ovarian masses, while AI and ML applications improve the accuracy of image interpretation. Proteomic and metabolomic studies reveal novel biomarkers and molecular insights. High-risk populations, often associated with genetic mutations such as BRCA1 and BRCA2, require specialized screening strategies. Current guidelines recommend screening modalities, risk-reduction strategies, and shared decision-making. Ongoing research focuses on refining risk assessment and personalized screening for high-risk individuals. This review underscores the importance of early detection in managing ovarian masses, emphasizing the need for improved screening methods, tailored approaches for high-risk populations, and ongoing research to further enhance diagnostic accuracy and patient outcomes.

## Introduction and background

Often encountered in clinical practice, ovarian masses encompass a diverse group of benign and malignant growths that develop within the ovaries. These masses can present many clinical challenges owing to their variable characteristics, ranging from small cysts with no symptoms to large tumors that can cause severe health complications. Ovarian masses affect women of all ages and can significantly impact their reproductive health, overall well-being, and, in some cases, their survival [1]. The classification of ovarian masses includes benign cysts, endometriomas, teratomas, and various types of ovarian cancer, each with unique clinical features and treatment requirements. Ovarian cancer represents a formidable adversary in women's health, as it is often diagnosed at an advanced stage, resulting in a poor prognosis. Early detection and timely intervention are crucial for improving outcomes and reducing the morbidity and mortality associated with ovarian masses [2].

Early detection of ovarian masses is vital in women's healthcare, as it offers the potential for more effective treatment and better overall patient outcomes. Unfortunately, the silent nature of many ovarian masses, especially in the initial stages, often leads to a delayed diagnosis. Consequently, most cases are identified at an advanced stage, limiting the treatment options and compromising the patient's quality of life [3]. Early detection facilitates less aggressive and more successful treatment and reduces the physical, emotional, and financial burden on individuals and healthcare systems. It is imperative to recognize the importance of proactive screening methods and diagnostic tools to identify ovarian masses in their early, treatable stages [4].

This comprehensive review aims to examine the current landscape of screening methods for ovarian masses and critically analyze their effectiveness, limitations, and prospects. We will delve into various diagnostic approaches, including imaging-based techniques such as transvaginal ultrasound, magnetic resonance imaging, computed tomography, blood-based biomarkers like CA-125, and emerging technologies including liquid biopsy and artificial intelligence applications.

## Review

Current diagnostic challenges

Limitations of Existing Screening Methods

Low sensitivity: Low sensitivity means that the current screening methods often fail to identify ovarian masses, especially in their early stages. Ovarian cancer is known as the "silent killer" as it frequently exhibits minimal or nonspecific symptoms until it reaches an advanced stage. Low sensitivity can result in false-negative results, where individuals with ovarian cancer receive a negative screening result, leading to a false sense of security, causing patients to delay seeking medical attention and potentially allowing the cancer to progress to more advanced and difficult-to-treat stages [5].

Specificity issues: The lack of specificity in screening methods means they are not very good at distinguishing between benign and malignant ovarian masses. Benign cysts or tumors can appear similar to cancerous ones in imaging or blood tests, leading to false-positive results. These false alarms can cause unnecessary stress, anxiety, and invasive diagnostic procedures, including surgery, which may not be required [6].

Inability to screen high-risk populations: Existing screening methods often do not identify ovarian cancer in high-risk populations, such as women with a family history of ovarian cancer or known genetic mutations like BRCA1 and BRCA2. These individuals have a significantly higher risk of developing ovarian cancer, but current screening methods may still miss the disease or provide inconclusive results [7].

Cost and accessibility: Some screening methods, such as regular ultrasound or blood tests (CA-125), can be expensive and not readily available to all individuals. The cost of screening and limited insurance coverage can create disparities in access to early detection. This inequity in access to screening can have dire consequences, as early diagnosis is critical for improving the prognosis for ovarian cancer [8].

Radiation exposure: Certain imaging-based screening methods, like computed tomography (CT) scans, involve ionizing radiation, which can be harmful with frequent screening. Excessive radiation exposure can increase the risk of other health issues. Limiting such methods as routine screening tools makes them less suitable for long-term, frequent monitoring, often necessary for high-risk individuals [9].

Diagnostic Delays and Their Consequences

Advanced-stage diagnoses: Delayed diagnosis resulting from the limitations of the current screening methods often leads to identifying ovarian masses at advanced stages of the disease. Ovarian cancer is most treatable when detected in its early stages. Advanced-stage diagnoses typically require more extensive and aggressive treatment, including debulking surgeries and aggressive chemotherapy. Unfortunately, the prognosis for advanced-stage ovarian cancer is significantly worse than early-stage cases. The chances of achieving complete remission and long-term survival are reduced, and the disease can be more challenging to manage [10].

Limited treatment options: Late-stage ovarian cancer diagnoses often limit the available treatment options. In advanced cases, the primary treatment typically involves aggressive surgical interventions to remove as much of the tumor as possible, followed by chemotherapy. The chances of successful treatment and long-term survival are diminished compared to cases diagnosed at earlier stages. Patients may also experience more severe side effects from treatment because of the advanced nature of the disease [11].

Increased healthcare costs: Treating advanced-stage ovarian masses is considerably higher than in early-stage cases. Late-stage diagnoses often require extended hospital stays, complex surgeries, and more aggressive chemotherapy regimens. These factors contribute to a significant financial burden on healthcare systems and patients. Furthermore, the long-term healthcare costs for advanced-stage cases can be substantial because of the ongoing treatment and care required to manage the disease [11].

Emotional and psychological distress: Diagnostic delays resulting from screening limitations can cause patients considerable emotional and psychological distress. The uncertainty of the diagnosis and the knowledge that the disease has progressed to an advanced stage can be emotionally devastating. Patients may experience anxiety, depression, fear, and uncertainty about their future. This emotional distress can significantly impact their overall quality of life, well-being, and ability to cope with the challenges of cancer treatment [12].

The Need for Improved Early Detection Strategies

Enhanced sensitivity and specificity: New screening methods must prioritize higher sensitivity and specificity. They should be capable of detecting ovarian masses accurately, particularly in their early stages. By improving sensitivity, these methods can help reduce false-negative results and enable earlier diagnosis. Additionally, enhanced specificity can reduce false-positive findings, preventing unnecessary anxiety and invasive diagnostic procedures [13].

Accessibility and affordability: Ensuring that early detection strategies are accessible to a broader population is crucial. These strategies should ideally be more cost-effective to reduce healthcare disparities. Affordable screening methods are essential to make early detection available to individuals from all socioeconomic backgrounds. Reducing the financial burden of screening can encourage more people to undergo regular testing [14].

Targeted screening for high-risk groups: It is essential to design strategies that effectively target high-risk populations, such as individuals with a family history of ovarian cancer or known genetic predispositions, such as BRCA1 and BRCA2 mutations. These individuals have a significantly elevated risk, and tailored screening protocols can improve early detection rates in these groups [15].

Reduced radiation exposure: Minimizing radiation exposure is crucial for the safety of individuals undergoing repeated screenings. New screening methods should focus on noninvasive and radiation-free techniques whenever possible. This consideration is significant for high-risk populations and long-term monitoring [16].

Integration of emerging technologies: Integrating emerging technologies, such as liquid biopsy and AI, can offer promising avenues for early detection. Liquid biopsy, for instance, can detect genetic material from tumors in the bloodstream, providing a less invasive and potentially susceptible method for cancer detection. AI can assist in interpreting complex imaging data, improving the accuracy and speed of diagnosis. Early detection strategies can benefit from these innovations by staying at the forefront of technological advancements [17].

Imaging-based screening methods

TVUS

TVUS plays a significant role in evaluating ovarian masses. This imaging modality involves the insertion of an ultrasound transducer into the vagina, allowing for high-resolution imaging of the pelvic organs. TVUS offers several notable benefits, contributing to its widespread use in clinical practice. It is a noninvasive procedure that is readily available and cost-effective. These qualities make it an accessible and practical tool for healthcare providers. TVUS is particularly valuable for visualizing ovarian cysts and providing detailed information about their size, morphology, and blood flow. Furthermore, it enables monitoring changes in ovarian masses over time, aiding in assessing disease progression [18].

However, TVUS also has certain limitations that need to be considered. It may have limited sensitivity in detecting small masses, especially in their early stages when they may be less conspicuous. Additionally, while TVUS can provide valuable information about the characteristics of ovarian masses, it may face challenges in differentiating between benign and malignant lesions based solely on imaging. To maximize the utility of TVUS, skilled sonographers and clinicians are essential for accurate image interpretation and clinical decision-making. Despite these limitations, TVUS remains a valuable tool for evaluating ovarian masses, offering a noninvasive and cost-effective approach for the initial assessment and monitoring of ovarian lesions [19].

Magnetic Resonance Imaging (MRI)

MRI is a powerful diagnostic tool for evaluating ovarian masses, providing detailed cross-sectional images of the pelvis with superior soft-tissue contrast. The procedure involves using strong magnetic fields and radio waves to generate high-resolution images. MRI serves various crucial applications in the assessment of ovarian masses. It is precious for characterizing complex ovarian lesions, enabling the differentiation between benign and malignant tumors, and facilitating cancer staging. Additionally, MRI effectively evaluates the extent of disease spread, aiding treatment planning and guiding surgical interventions [20].

One of the critical advantages of MRI is its excellent sensitivity and specificity, which allow for precise and accurate characterization of ovarian masses. It is a noninvasive imaging technique that provides detailed information about the structural and functional aspects of the pelvic organs. MRI is especially beneficial when TVUS results are inconclusive or when a more comprehensive evaluation of ovarian masses is necessary to make informed clinical decisions [21].

However, certain limitations associated with MRI need to be considered. The cost of MRI can be substantial, making it a relatively expensive imaging modality compared to TVUS. Additionally, MRI may not be as widely available as TVUS in all healthcare settings, potentially limiting its accessibility for some patients. Moreover, MRI may not be suitable for individuals with certain medical conditions or contraindications for magnetic resonance imaging, such as pacemakers, metallic implants, or severe claustrophobia. Despite these limitations, MRI remains an invaluable tool in the comprehensive evaluation of ovarian masses, offering precise and detailed insights that can guide clinical management decisions and improve patient outcomes [22].

Computed Tomography (CT)

CT is an imaging modality that employs X-rays and computer processing to generate cross-sectional images of the body, which can be informative for assessing the location and characteristics of ovarian masses. CT scans have specific applications in cases where ovarian masses are associated with other abdominal or pelvic conditions, providing valuable insights into the extent of the disease and aiding in surgical planning [9]. One of the primary benefits of CT is its ready availability and rapid results. CT scans can quickly and efficiently provide detailed information about the anatomy and pathology of the abdomen and pelvis, which can be valuable when ovarian masses are suspected to be part of a broader clinical picture. CT can also assist in identifying secondary effects of ovarian masses, such as lymph node involvement or distant metastases, which is crucial for staging and treatment planning [23].

However, CT does come with limitations that must be considered. One of the main limitations is its exposure to ionizing radiation, which can be a concern, particularly for patients undergoing repeated screenings. This radiation exposure may limit CT as a routine screening tool, especially when safer alternatives like TVUS are available. CT is also less sensitive in detecting small lesions and may not be the preferred choice for the initial evaluation of ovarian masses. Instead, it is typically used in cases with suspected complications or to assess the broader clinical context. Overall, while CT has essential applications in certain situations, its limitations, including radiation exposure and reduced sensitivity for small lesions, should be carefully weighed against its benefits when considering its use in evaluating ovarian masses [24].

Positron Emission Tomography (PET) Scans

PET scans play a significant role in detecting and staging ovarian cancer. The procedure involves the administration of a radioactive tracer, which accumulates in areas of increased metabolic activity, such as cancer cells. The distribution of this tracer is then imaged to provide valuable insights into the metabolic activity of tissues [25]. PET scans have several essential applications in the assessment of ovarian cancer. They can aid in detecting ovarian cancer by identifying areas of increased metabolic activity, helping to determine the extent of the disease and its spread. PET scans are often combined with other imaging modalities to comprehensively evaluate the disease, contributing to more accurate staging and treatment planning [26].

One of the critical benefits of PET scans is their ability to help differentiate between benign and malignant masses, offering valuable information for treatment decision-making and monitoring therapy effectiveness. By visualizing the metabolic activity within the tissues, PET scans can provide crucial information about the aggressiveness and spread of the cancer, guiding clinicians in devising appropriate treatment strategies [27]. However, PET scans have certain limitations that need to be considered. They may not always distinguish between malignant masses and certain nonmalignant conditions, potentially leading to false-positive or false-negative results. Additionally, the radiation exposure associated with PET scans is a relevant factor, particularly when considering repeated or frequent imaging. Cost considerations also play a role, as PET scans can be relatively expensive compared to other imaging modalities [28].

Emerging Technologies and Advances in Imaging

Emerging technologies: Ongoing research is investigating various advanced imaging techniques, such as functional MRI (fMRI), diffusion-weighted imaging (DWI), and dynamic contrast-enhanced MRI (DCE-MRI), to enhance the accuracy of ovarian mass evaluation. These techniques can provide valuable insights into the characteristics and behavior of ovarian masses, aiding in the differentiation between benign and malignant lesions. Functional MRI, for example, can assess blood flow and tissue perfusion, while DWI can help detect cellular density variations [29].

AI: AI and machine learning (ML) play a significant role in improving the accuracy of ovarian mass assessment. AI algorithms can analyze complex imaging data and assist in the early detection and characterization of ovarian masses. They can recognize patterns, identify subtle features that may be missed by human observers, and provide quantitative data to aid in diagnosis. AI-driven image analysis has the potential to reduce both false-negative and false-positive results [30].

Advancements in ultrasound: Ultrasonography, particularly TVUS, is a valuable tool in ovarian mass assessment. Advancements in ultrasound technology, including 3D and 4D ultrasound, contrast-enhanced ultrasound, and elastography, have enhanced the capabilities of TVUS. These innovations provide more detailed and real-time information about ovarian masses, improving the accuracy of diagnosis. Contrast-enhanced ultrasound, for example, can highlight blood flow patterns in tumors, while elastography can assess tissue stiffness, aiding in the differentiation of benign and malignant masses [31].

Image-guided biopsies: The integration of imaging with image-guided biopsies is a significant advancement in ovarian mass assessment. This approach allows for more precise and targeted sampling of suspicious ovarian masses, leading to accurate diagnosis and facilitating treatment planning. Image-guided biopsies, such as ultrasound-guided or MRI-guided, can reduce the likelihood of inadequate or inconclusive results, ensuring that the obtained tissue samples represent the lesion in question [32].

Blood-based biomarkers

CA-125 and Its Limitations

Sensitivity and specificity: CA-125 lacks the necessary sensitivity and specificity to be a reliable standalone diagnostic tool. Sensitivity refers to its ability to accurately detect actual cases of ovarian cancer, while specificity pertains to its ability to identify individuals without the disease correctly. CA-125's sensitivity is limited, as elevated levels can occur in various benign conditions and may not always correlate with ovarian cancer. Consequently, it can result in false-positive and false-negative results [33].

False positives and negatives: CA-125 can produce false-positive results when elevated levels are observed in benign conditions, such as pelvic inflammatory disease or endometriosis. Conversely, it can produce false-negative results, especially in the early stages of ovarian cancer, when the levels may remain within the normal range. These inaccuracies can complicate the diagnostic process, leading to unnecessary tests, patient anxiety, and potentially missed diagnoses [34].

Non-specificity: CA-125 is not specific to ovarian cancer; elevated levels can also be seen in other gynecological malignancies (e.g., uterine or fallopian tube cancer) and non-gynecological cancers (e.g., pancreatic or lung cancer). This lack of specificity can further confound the interpretation of CA-125 levels in clinical practice [34].

Serial monitoring: While CA-125 has some clinical utility, it is often used for serial monitoring of levels rather than as a one-time screening tool. This means that multiple tests are needed over time to observe trends in CA-125 levels. The requirement for serial monitoring can delay the diagnosis of ovarian cancer, which is a concern as early detection is crucial for improving outcomes [35].

Variability: There is interpatient and intraindividual variability in CA-125 levels. This variability can make it challenging to establish clear cutoff values for the biomarker and complicate its interpretation. Additionally, factors like inflammation or benign conditions can transiently elevate CA-125 levels, further complicating the assessment of results [36].

Other Potential Serum Biomarkers

Human epididymis protein 4 (HE4): HE4 is a protein that has gained attention as a valuable biomarker for ovarian cancer detection. It is often combined with CA-125 to improve the sensitivity and specificity of ovarian cancer diagnosis. HE4 can be elevated in the early stages of the disease, providing complementary information to CA-125. Its ability to detect early-stage disease is particularly significant, as early detection is critical for improving treatment outcomes. HE4 is known for its potential to distinguish between benign and malignant ovarian masses, reducing the number of false-positive and false-negative results. Its integration into diagnostic protocols has shown promise in enhancing the accuracy of ovarian cancer detection and improving patient outcomes [37].

Risk of ovarian malignancy algorithm (ROMA): ROMA is a sophisticated algorithm that combines the use of CA-125 and HE4 levels, along with the consideration of menopausal status, to enhance the accuracy of ovarian cancer detection. By utilizing multiple parameters, including the levels of both biomarkers and the patient's menopausal status, ROMA improves the reliability of assessing the risk of ovarian malignancy. This algorithm aids healthcare providers in making informed decisions regarding the need for further evaluation and referral to specialists. By considering a combination of factors, ROMA can potentially reduce the rate of misdiagnosis and provide more accurate risk assessment for patients with suspected ovarian cancer [38].

OVA1: OVA1 is a multi-marker assay that goes beyond CA-125 alone. It combines CA-125 with four additional biomarkers to comprehensively assess ovarian cancer risk. By incorporating multiple biomarkers, OVA1 provides a more nuanced evaluation of the likelihood of ovarian cancer, allowing healthcare providers to make more informed decisions regarding the necessity of referral to a specialist. OVA1's multi-marker approach improves the accuracy of risk assessment, aiding in the early detection and management of ovarian cancer and reducing unnecessary surgical interventions and associated healthcare costs [39].

MicroRNA: Certain microRNAs, such as miR-200 and miR-214, have shown potential as blood-based biomarkers for ovarian cancer. These small non-coding RNA molecules play crucial roles in gene expression regulation and have been identified as potential indicators of ovarian cancer. MicroRNAs, including miR-200 and miR-214, are being extensively investigated for their diagnostic and prognostic value. Their presence and expression patterns in the blood can provide valuable information on the development and progression of ovarian cancer, potentially enabling earlier detection and more accurate monitoring of the disease [40].

Exosome-based biomarkers: Exosomes are small extracellular vesicles released by various cells, including tumor cells, into bodily fluids such as blood or urine. These vesicles contain various biomolecules, including proteins, nucleic acids, and lipids, which can be analyzed for their diagnostic potential. Exosome-based biomarkers are emerging as a promising avenue for detecting ovarian masses. The analysis of exosomal contents can provide valuable insights into the molecular characteristics of ovarian tumors, aiding in the early detection, classification, and monitoring of the disease. The use of exosome-based biomarkers holds promise for improving the accuracy and reliability of ovarian cancer diagnosis and may lead to more effective personalized treatment strategies [41].

Role of Proteomics and Genomics in Biomarker Discovery

Proteomics: Proteomics studies the complete set of proteins within a biological sample, such as blood. In ovarian cancer, proteomic studies systematically analyze proteins in blood samples to identify specific biomarkers associated with ovarian masses. This approach helps researchers and clinicians understand the protein expression patterns and alterations that may indicate the presence of ovarian cancer. High-throughput techniques (e.g., mass spectrometry) are pivotal in proteomic research for accurately identifying and quantifying proteins. By analyzing the protein profile in the blood, proteomics can reveal potential diagnostic or prognostic markers that can aid in early detection and personalized treatment strategies [42].

Genomics: Genomics analyzes genetic information encoded within DNA and RNA. In the context of ovarian cancer, genomic approaches aim to identify genetic markers, mutations, and variations associated with the disease. By sequencing and analyzing the genetic material, researchers can pinpoint specific genetic changes that increase the risk of ovarian cancer or influence treatment responses. Genomic insights can also guide the development of targeted therapies tailored to the tumor's genetic makeup. This approach provides a foundation for risk assessment, early detection, and developing personalized treatment plans for ovarian cancer patients [43].

Integrated omics approaches: Researchers are increasingly adopting integrated omics approaches to obtain a more comprehensive understanding of ovarian cancer at the molecular level. These approaches combine data from multiple omics fields, including proteomics, genomics, and metabolomics. By integrating these diverse sources of information, researchers can uncover complex interactions between proteins, genes, and metabolic pathways associated with ovarian masses. This holistic approach can potentially identify more accurate and reliable biomarkers for early detection and personalized treatment. Integrated omics approaches also enhance our understanding of the disease's underlying mechanisms, paving the way for developing more effective therapies [44].

Ultrasound-based scoring systems

Risk of Malignancy Index (RMI)

RMI is a widely utilized scoring system for evaluating ovarian masses. It integrates TVUS findings and serum CA-125 levels to estimate the likelihood of malignancy. The RMI incorporates multiple components to generate a numerical score that assists healthcare professionals in determining the need for further evaluation and management [45]. The RMI's key components typically include assessing the mass through transvaginal ultrasound, measuring serum CA-125 levels, and considering the patient's menopausal status. Various versions of the RMI exist, with different guidelines and scoring criteria utilized by different institutions and healthcare providers. The combination of these components provides valuable information for risk stratification, aiding in clinical decision-making and facilitating appropriate referrals for surgical evaluation when necessary [46].

While the RMI serves an essential purpose in estimating the risk of malignancy associated with ovarian masses, it has notable limitations that must be considered. The RMI has been criticized for its reduced sensitivity in detecting early-stage malignancies, potentially leading to false-negative results and delayed diagnoses. Moreover, the RMI may only partially encompass the broader clinical context and individual patient factors that could influence the interpretation of results. Healthcare professionals should be aware of these limitations when applying the RMI and consider supplementary diagnostic tools and assessments to ensure comprehensive and accurate risk evaluation in managing ovarian masses [47].

Ovarian-Adnexal Reporting and Data System (O-RADS)

O-RADS is a standardized framework designed to report and characterize ovarian masses using ultrasound imaging comprehensively. It provides a structured approach to categorize and assess these masses based on specific ultrasound features and morphology [48]. At its core, O-RADS categorizes ovarian masses into distinct risk groups, such as O-RADS 1 for benign lesions and O-RADS 5 for malignant or highly suspicious lesions. These categories are determined based on the presence or absence of various ultrasound features, including the size, shape, internal structure, vascularity, and other specific mass characteristics [49].

The primary purpose of O-RADS is to establish a common language and standardized reporting system that enables consistent communication among healthcare providers. By facilitating uniformity in reporting, O-RADS streamlines the interpretation of ultrasound findings and aids in the accurate risk assessment of ovarian masses. This standardized approach contributes to more informed clinical decision-making, guiding appropriate management strategies and interventions [50]. One of the critical benefits of O-RADS is its user-friendly nature, which allows healthcare professionals to describe and classify ovarian masses according to standardized criteria quickly and effectively. By reducing ambiguity in reporting and interpretation, O-RADS helps clinicians determine the need more confidently for further evaluation, such as additional imaging studies or surgical interventions [51].

O-RADS has gained significant recognition and endorsement from various professional organizations in gynecology and radiology. Its implementation is encouraged in ultrasound reporting and research, aiming to improve the consistency and reliability of ovarian mass characterization across different healthcare settings. As a standardized and widely accepted framework, O-RADS is crucial in enhancing the quality and efficiency of ovarian mass assessment and management [52].

International Ovarian Tumor Analysis (IOTA) Group

IOTA group is a collaborative network comprising researchers and clinicians dedicated to advancing the ultrasound-based characterization of ovarian masses. The group has significantly contributed to gynaecology and radiology by developing risk prediction models, standardized terms, and definitions for ultrasound features related to ovarian masses [53]. Critical components of IOTA's work include the development of risk prediction models, such as the IOTA Simple Rules. These models offer clear guidelines for clinicians and sonographers to classify ovarian masses as benign or malignant based on specific ultrasound features. IOTA aims to enhance the accuracy of ultrasound diagnosis for ovarian masses, ultimately reducing unnecessary surgeries and improving patient care [54].

The benefits of IOTA's efforts are substantial. Research has shown that IOTA's models and guidelines can significantly improve the specificity and sensitivity of ultrasound in distinguishing between benign and malignant masses. By providing a structured approach to the ultrasound-based assessment of ovarian masses, IOTA's contributions offer a valuable resource for clinicians and sonographers, aiding in more accurate and consistent diagnoses [55]. IOTA researches to refine its models and guidelines and offers training programs to promote their widespread and consistent use in clinical practice. Through research and education, IOTA plays a pivotal role in improving the quality of ovarian mass assessment, enhancing patient outcomes, and reducing the burden of unnecessary surgical procedures for patients [56].

Emerging technologies

Liquid Biopsy and Circulating Tumor DNA

Liquid biopsy is an innovative approach that involves the analysis of biological fluids, such as blood, to detect the presence of circulating tumor DNA (ctDNA) and other biomarkers shed by ovarian masses. This technique has significant potential in ovarian cancer diagnosis and management [57]. The applications of liquid biopsy in ovarian cancer are wide-ranging. It can be employed for early detection, monitoring the response to treatment, and assessing the progression of the disease. What sets liquid biopsy apart is its minimally invasive nature, which allows for real-time evaluation of genetic changes in the tumor. Unlike traditional tissue biopsies, which can be invasive and may not be suitable for frequent monitoring, liquid biopsy provides a less burdensome method for tracking the genetic profile of ovarian masses [58].

One of the most compelling benefits of liquid biopsy is the opportunity for more frequent and dynamic monitoring of ovarian cancer can lead to earlier detection of disease recurrence or the development of treatment resistance, enabling healthcare providers to adjust their treatment strategies promptly. Furthermore, liquid biopsy can help tailor therapies to the evolving genetic profile of the tumor, optimizing the choice of treatments based on the tumor's specific genetic changes [59]. However, there are challenges associated with implementing liquid biopsy for ovarian masses. The levels of ctDNA in the bloodstream can be quite low, making detection more challenging, particularly in early-stage disease, which can result in potential false negatives. Additionally, there is a need for standardized protocols and further research to establish the reliability and clinical utility of liquid biopsy in ovarian cancer diagnosis and management [60].

AI and ML Applications

AI and ML are instrumental in developing algorithms and models that can significantly contribute to diagnosing and characterizing ovarian masses [61]. The applications of AI and ML in this context are wide ranging. These technologies can enhance the accuracy of image interpretation, such as the analysis of ultrasound, MRI, or CT scans. AI and ML algorithms can identify subtle patterns and features that human observers may miss. They can also assist in the classification of ovarian masses as either benign or malignant, offering a more objective and data-driven approach to diagnosis [62].

One of the key benefits of AI and ML in the context of ovarian mass evaluation is their potential to improve the sensitivity and specificity of existing screening and diagnostic methods. By leveraging large datasets and sophisticated algorithms, these technologies can reduce subjectivity in interpretation and provide real-time decision support for healthcare providers, helping them make more informed clinical decisions [63]. Despite the promise of AI and ML, notable challenges are associated with their implementation in clinical practice. One significant challenge is the requirement for large and diverse datasets for training and validating these algorithms. Another concern is the potential for bias in algorithm development, which must be carefully addressed to ensure equitable and accurate results for all patient groups. Regulatory approval is also a crucial step, as AI and ML applications in healthcare need to meet rigorous standards of safety and effectiveness before they can be integrated into clinical practice [64].

Proteomic and Metabolomic Approaches

Proteomics involves the comprehensive analysis of proteins in biological samples, while metabolomics studies metabolites, providing insights into the molecular processes and pathways associated with ovarian masses. Both approaches aim to identify specific markers that can aid in the early detection and classification of these masses [65]. The applications of proteomic and metabolomic studies are broad and multifaceted. They are employed to discover novel biomarkers that can enhance the sensitivity and specificity of ovarian mass detection. These approaches can also offer a deeper understanding of the molecular pathways and mechanisms underlying ovarian masses, shedding light on the disease's biology and potential therapeutic targets [66].

One of the main potential benefits of proteomic and metabolomic research is the discovery of new biomarkers that can significantly improve our ability to detect and classify ovarian masses accurately. These approaches promise better diagnostic tools by identifying markers with enhanced sensitivity and specificity. Moreover, the insights gained from proteomic and metabolomic studies can provide a more profound understanding of the molecular intricacies of ovarian masses, potentially opening doors to targeted therapies and personalized treatment strategies [67]. However, there are challenges associated with proteomic and metabolomic approaches. The data generated through these methods are often complex and high-dimensional, necessitating robust analytical techniques for interpretation. Additionally, newly identified biomarkers must be rigorously validated in large patient cohorts to ensure their reliability and clinical utility [68].

Screening in high-risk populations

Genetic Predispositions and High-Risk Groups

Genetic mutations: High-risk populations often encompass individuals with well-documented genetic mutations, most notably BRCA1 and BRCA2. These mutations, associated with hereditary breast and ovarian cancer syndrome (HBOC), significantly elevate the risk of developing ovarian cancer. Those with BRCA1 mutations face a lifetime risk of about 44% to 17% for BRCA2 mutations, which is markedly higher than the general population's risk. Thus, individuals carrying these mutations are prime candidates for inclusion in high-risk groups [7].

Family history: A strong family history is pivotal in identifying high-risk individuals. Individuals with close relatives (e.g., mother, sister, daughter) diagnosed with ovarian cancer or related cancers, mainly if multiple cases exist within a family, are at increased risk. Such family histories may trigger heightened vigilance and consideration for high-risk group status [69].

Other risk factors: Besides well-established genetic mutations and family history, other risk factors contribute to high-risk status. Lynch syndrome, which is linked to a higher risk of colorectal and endometrial cancers, may also elevate the risk of ovarian cancer. Lynch syndrome and similar conditions associated with a genetic predisposition to multiple cancers can place individuals in high-risk groups [70].

Increased surveillance: High-risk populations necessitate intensified surveillance strategies to facilitate early detection of ovarian masses when they are more amenable to treatment. Enhanced surveillance may involve more frequent and in-depth screening modalities, often beginning at an earlier age. These strategies aim to identify ovarian masses at an earlier, more treatable stage, improving patient outcomes and reducing the morbidity and mortality associated with ovarian cancer [71].

Current Guidelines and Recommendations

Clinical guidelines: Several professional organizations, including the American College of Obstetricians and Gynecologists (ACOG), the National Comprehensive Cancer Network (NCCN), and the Society of Gynecologic Oncology (SGO), have developed clinical guidelines and recommendations for screening in high-risk populations. These guidelines are essential reference points for healthcare providers and are continually updated to reflect the latest evidence-based practices in ovarian mass screening [72].

Screening modalities: Current guidelines often include specific recommendations regarding the screening modalities suitable for high-risk individuals. These may encompass TVUS and serum CA-125 measurements, both commonly employed methods. The guidelines may also stipulate the optimal age for screening and the intervals between screening sessions. These parameters are carefully considered to maximize the effectiveness of early detection [73].

Risk-reducing strategies: In individuals with high genetic risk, guidelines may advocate for risk-reduction strategies. One such approach is prophylactic surgery, known as salpingo-oophorectomy, where the fallopian tubes and ovaries are surgically removed. This strategy can substantially reduce the risk of developing ovarian masses and cancer. However, the decision to undergo such procedures is typically guided by individual risk assessments and personal preferences [74].

Shared decision-making: Guidelines consistently emphasize the importance of shared decision-making between healthcare providers and patients in the context of ovarian mass screening for high-risk individuals. The shared decision-making process recognizes that each patient is unique, and their preferences and values play a significant role in determining the most suitable screening approach. Healthcare providers engage in informed discussions with patients, considering their risk profiles and addressing their specific concerns and preferences to make collective decisions about screening and risk-reduction strategies [75].

Ongoing Research and Future Directions

Improved risk assessment: Ongoing research enhances risk assessment for high-risk populations by identifying additional genetic markers and risk factors. Beyond established genetic mutations like BRCA1 and BRCA2, researchers are exploring the contribution of other genetic alterations and environmental factors to ovarian cancer risk. This comprehensive approach to risk assessment allows for a more precise understanding of an individual's likelihood of developing ovarian masses, leading to more tailored and effective screening strategies [76].

Personalized screening: The future of screening in high-risk groups is moving towards personalized approaches. Not all high-risk individuals have identical genetic and familial risk profiles, so personalized screening regimens are becoming increasingly important. This approach considers an individual's genetic mutations, familial history, and other risk factors. As a result, screening plans are tailored to match the unique risk profile of each person, optimizing the accuracy and effectiveness of early detection efforts [77].

Emerging technologies: Integrating emerging technologies is poised to revolutionize screening in high-risk populations. Liquid biopsy, for instance, offers a minimally invasive method for the real-time monitoring of genetic alterations in circulating tumor DNA, enhancing the sensitivity and specificity of ovarian mass detection. AI and ML applications are expected to play a pivotal role in identifying subtle patterns and nuances in imaging data, enabling more precise risk assessment [78].

Clinical trials: Clinical trials are at the forefront of innovation in screening high-risk populations for ovarian masses. Researchers actively investigate new screening modalities and risk-reduction strategies to advance early detection and management. These trials seek to validate the efficacy of novel technologies and approaches, ultimately improving the lives of high-risk individuals by identifying ovarian masses at earlier, more treatable stages [79].

Psychosocial support: Future directions in ovarian mass screening for high-risk populations should extend beyond the clinical and technical aspects. Recognizing the psychosocial challenges faced by high-risk individuals, the integration of psychosocial support is essential. This includes counseling services and emotional support to help individuals navigate the emotional and decision-making aspects of screening and risk reduction. It addresses high-risk populations' unique psychological and emotional needs, providing a holistic approach to care [80].

## Conclusions

The review of ovarian mass screening and evaluation highlights the need for improved methods because of existing limitations, including delayed diagnoses and their associated consequences. Promising advancements include innovative imaging techniques, such as MRI, the integration of emerging technologies like AI and liquid biopsy, and standardized reporting systems like O-RADS and IOTA guidelines. These innovations have the potential to enhance early detection, reduce invasive treatments, and improve patient outcomes. Looking forward, the field will focus on refining AI and ML algorithms, exploring novel biomarkers, and adopting multidisciplinary approaches, ultimately aiming to provide more accurate, personalized, and minimally invasive strategies for assessing and managing ovarian masses. These developments hold the promise of significantly improving the well-being of individuals at risk of ovarian cancer.
